# A Method for Grouping Emergency Department Visits by Severity and Complexity

**DOI:** 10.5811/westjem.2020.6.44086

**Published:** 2020-08-21

**Authors:** B. Jason Theiling, Kendrick V. Kennedy, Alexander T. Limkakeng, Pratik Manandhar, Alaatin Erkanli, Stephen R. Pitts

**Affiliations:** * Duke University School of Medicine, Department of Emergency Medicine, Durham, North Carolina; † Duke University School of Medicine, Department of Biostatistics and Bioinformatics, Durham, North Carolina; ‡ Emory University School of Medicine, Department of Emergency Medicine, Atlanta, Georgia

## Abstract

**Introduction:**

Triage functions to quickly prioritize care and sort patients by anticipated resource needs. Despite widespread use of the Emergency Severity Index (ESI), there is still no universal standard for emergency department (ED) triage. Thus, it can be difficult to objectively assess national trends in ED acuity and resource requirements. We sought to derive an ESI from National Hospital Ambulatory Medical Care Survey (NHAMCS) survey items (NHAMCS-ESI) and to assess the performance of this index with respect to stratifying outcomes, including hospital admission, waiting times, and ED length of stay (LOS).

**Methods:**

We used data from the 2010–2015 NHAMCS, to create a measure of ED visit complexity based on variables within NHAMCS. We used NHAMCS data on chief complaint, vitals, resources used, interventions, and pain level to group ED visits into five levels of acuity using a stepwise algorithm that mirrored ESI. In addition, we examined associations of NHAMCS-ESI with typical indicators of acuity such as waiting time, LOS, and disposition. The NHAMCS-ESI categorization was also compared against the “immediacy” variable across all of these outcomes. Visit counts used weighted scores to estimate national levels of ED visits.

**Results:**

The NHAMCS ED visits represent an estimated 805,726,000 ED visits over this time period. NHAMCS-ESI categorized visits somewhat evenly, with most visits (42.5%) categorized as a level 3. The categorization pattern is distinct from that of the “immediacy” variable within NHAMCS. Of admitted patients, 89% were categorized as NHAMCS-ESI level 2–3. Median ED waiting times increased as NHAMCS-ESI levels decreased in acuity (from approximately 14 minutes to 25 minutes). Median LOS decreased as NHAMCS-ESI decreased from almost 200 minutes for level 1 patients to nearly 80 minutes for level 5 patients.

**Conclusion:**

We derived an objective tool to measure an ED visit’s complexity and resource use. This tool can be validated and used to compare complexity of ED visits across hospitals and regions, and over time.

## INTRODUCTION

In the current practice of emergency medicine, triage functions to quickly prioritize care and sort patients by anticipated resource needs. While the goal of accurate prioritization is direct improvement in the quality of care of individuals, the intention of predicting resource utilization is to streamline emergency department (ED) operations without causing harm. The second goal has become increasingly important as the number of ED visits continues to rise, hospitals function under reduced available capacity, and reliance on the ED as the safety net of hospital systems increases.^1^ This dual function of ED triage was proposed by Wuerz, who pioneered a five-level Emergency Severity Index (ESI).^2^ Several studies have evaluated ESI’s reliability and validity.^2^–^10^ Other five-level triage schemes have been developed outside the United States: the Canadian Triage and Acuity Scale (CTAS),^11^–^12^ and the Australasian Triage Scale (ATS).^13^ Both CTAS and ATS assign each triage level a target “time to be seen,” which in turn allows comparisons between sites on the basis of compliance with these targets. The ATS is unique in explicitly accepting a third role as a data source for describing case-mix to generate the adjusted estimates of ED visit characteristics that inform national policy.^12^

Comparing ED performance and ED visit characteristics is more problematic in the US. Despite geographic variation in important ED characteristics (eg, the proportion of safety-net visits,^14^ population-based ED visit rates,^15^ and hospital admission rates^16^) there is still no national mandated standard for categorizing the acuity and resource complexity of ED visits. Central to the ESI system is the idea of “immediacy,” a marker of how acutely ill a patient is believed to be and thus how “immediately” they may need to be seen. Unfortunately, because of the continued widely disparate triage procedures and non-response in surveys, adjustment of ED data on the basis of the “immediacy” item alone is potentially biased and may ignore the dimension of care complexity. Additionally, the acuity or “immediacy” of a patient, as denoted by ESI, often does not linearly correlate with resource utilization during that ED visit.

The purpose of this study was to evaluate the “immediacy” variable in existing National Hospital Ambulatory Medical Care Survey (NHAMCS) survey data, and to create a practical alternative method for grouping ED visits by both acuity and resource complexity in a manner analogous to the ESI. To minimize data loss we sought to derive an ESI from NHAMCS survey items with low frequencies of item non-response. We assessed the performance of this index with respect to several outcomes, including hospital admission, waiting time, and overall ED length of stay (LOS).

This study uses the combined 2010–2015 ED components of the NHAMCS.^16^ The NHAMCS is a probability sample of US hospital EDs and outpatient departments conducted annually since 1992. It is one of a family of healthcare surveys performed by the Centers for Disease Control and Prevention’s (CDC) National Center for Health Statistics (NCHS). The US Census Bureau is responsible for field operations and data collection. Although one of its data items is currently a five-level item called “immediacy with which patient should be seen,” with additional checkboxes for “no triage” and “unknown,” other measures of urgency have been abstracted from ED charts in the past. From 1992–1996 the survey captured a highly subjective two-level “Urgent/emergent vs. Non-urgent” item, which led to the widely cited and heavily criticized conclusion that “55% of ED visits are non-urgent.”^17^ In 1997 this item was replaced by a four-level variable to capture more degrees of immediacy, each succeeding level associated with a progressively longer target “time to be seen.” In 2005 “immediacy” was promoted to the current five-level item, each level again associated with target times.

**Population Health Research Capsule**

What do we already know about this issue?

Triaging prioritizes care and sorts patients by anticipated resource needs. Despite widespread use of the Emergency Severity Index (ESI), no universal standard exists.

What was the research question?

Derive an ESI tool from a national survey item and assess the performance of this index with respect to stratifying outcomes.

What was the major finding of the study?

This tool can be used to compare complexity of ED visits across hospitals and regions, and over time.

How does this improve population health?

ESI may not reflect resource needs in linear fashion. Our tool helps to compare data across regions and time periods.

## METHODS

The NHAMCS is a four-stage probability sample, sampled in the following sequence: 1) 112 geographic primary sampling units of approximately county size; 2) probability sample of nonfederal, short-stay, general hospitals with EDs or outpatient departments or both, within the sampled primary sampling units, selected from a publicly available database of all US hospitals; 3) emergency service areas within 24-hour EDs and clinics within outpatient departments; and 4) a sample of about 100 visits within the selected EDs or outpatient departments during a randomly assigned four-week reporting period throughout the year. We limited our analysis to the ED component of NHAMCS and downloaded data from the NHAMCS website (*ftp://ftp.cdc.gov/pub/Health_Statistics/NCHS/Datasets/NHAMCS)*. Hospital staff were asked to complete a patient record form (PRF) for a sample of visits during a four-week reporting period, from which the data were abstracted and coded. The NHAMCS was approved by Duke University Insitutational Review Board. A report published elsewhere describes the plan and operation of the NHAMCS in greater detail.^17^ Unless otherwise noted, all estimates in this report are weighted to give national estimates. We considered estimates based on an unweighted count of less than 30 to be unreliable.

### Creating The NHAMCS-ESI Index

We based the NHAMCS-ESI (ESI-N) on the published ESI,^2^ but used only variables available in NHAMCS (Table 1). Since ESI and other tools are used in the initial triage process, they are dependent on data available immediately upon or shortly after presentation. Thus, ESI-N uses the presenting complaint rather than the final diagnosis as the main component. For NHAMCS-ED, this complaint is abstracted directly from the actual ED chart into up to three free-text entry fields on the PRF. The PRFs are then batched, and the hand-written text is converted to standard codes by the Constella Group, Inc. (Durham, NC). According to the *reason for visit classification for ambulatory care* (RVC), there is a very low rate (<1%) of nonresponse. Additionally, vital signs have been recorded since 2001 and can be used to modify triage class just as the ESI does. Vital signs are not obtained on every visit, especially among the pediatric population; thus, when vital signs were missing, we considered the ESI-N unchanged rather than missing. Unlike the ESI, the retrospective ESI-N tallies actual resources used, rather than predicted resources, and cannot account for any resources not listed in the PRF.

### Describing The Acuity Of The Patients And Validating The Index

We described the acuity of the study population by generating basic descriptive statistics including the mean ESI-N level and corresponding confidence intervals (CI) for selected patient and hospital characteristics. To validate the ESI-N, we examined associations with typical indicators of acuity such as waiting time, LOS, disposition, and mode of arrival. Waiting time was defined as the number of minutes between the time of arrival and the time seen by a physician. We defined LOS as the number of minutes between the time of arrival and time of ED discharge. All of these outcomes measures of acuity (waiting time, LOS, disposition, and mode of arrival) were measured in their respective units or proportions for each level of the derived ESI-N and compared using 95% CIs. We conducted all analyses using Stata v10 (StataCorp, College Station, TX).^19^

## RESULTS

The “immediacy” item in NHAMCS-ED (IMMED) was unknown, or triage was not performed, in 15.0% of visits. Among the remaining visits, the maximum value of IMMED was less than five in 145 (26.2%) of 553 emergency service areas in the 457 hospitals surveyed. This suggests the continued use of triage schemes with fewer than five levels. Unfortunately, having four or fewer triage levels creates a bias toward lower (more acute) immediacy levels when incorporated in aggregate estimates.

**Figure 1:**
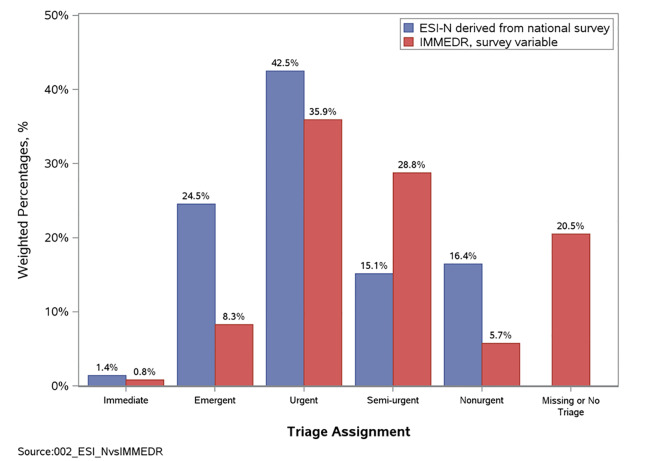
Distribution of Emergency Severity Index levels based on the presenting complaint. *IMMEDR*, immediacy.

These results are given both in raw counts of 2010–2015 patient record forms, and in a nationally representative estimate of percent of all visits. Figure 1 compares this frequency distribution both to the distribution of the variable IMMED in the same data, and to the distribution of actual ESI levels assigned by triage nurses in a prospective validation study of two ED populations.^20^

We assessed mean ESI levels for a variety of patient characteristics, as demonstrated in Table 2. For each of the four patient-level characteristics that we assessed (age, gender, race/ethnicity, and payer type), there were statistically significant differences (p<0.0001) between mean ESI-N levels. Of note, there was a monotonic increase in acuity with increasing age. Within their respective categories, visits by women and by non-Hispanic Whites had on average more acute ESI-N scores. Among payer types, Medicare visits had on average the most acute ESI-N, with the least acute categories being worker’s compensation, “no charge,” and Medicaid/State Children’s Hospital Insurance Program.

We assessed validity of the ESI-N against several outcomes, including hospital admission, intensive care unit (ICU) admission, ambulance arrival, and leaving without being seen and in each case found the expected relationships (Table 3). As can be seen, the ESI-N is able to differentiate all visits into five levels of care, with only a minority being classified in the most severe, “Immediate” category. Relative to their proportion of total visits, more severe acuity ESI-N levels account for a higher proportion of the patients admitted to the hospital and those admitted to a critical care unit.

Furthermore, ESI-N is able to differentiate visits (Table 4) by likelihood of being admitted to the hospital and ED LOS. The 95% CIs of percent admitted or LOS in minutes between categories largely do not overlap as the ESI-N increases in severity from Level 5 “Nonurgent” to Level 1 “Immediate.” ESI-N does not appear to differentiate well among categories for time waiting to be seen by a physician, however.

## DISCUSSION

The NHAMCS-ED is the only nationally representative survey of ED visits; thus, it is a valuable resource for policy-making and has provided data for many published studies. However, most causal inferences of interest, such as the effect of demographic variables like race/ethnicity on hospital admission, length of ED visit, and other aspects of quality and cost, are potentially confounded by the main determinant of these outcomes: the seriousness or acuity of the patient’s initial problem. A related reason to measure acuity is the need for fair comparisons across regions or over time, i.e., for case-mix adjustment. We have derived a five-level index of acuity called the ESI-N, because a similar existing item in the survey, called “immediacy,” frequently offered difficulties in chart abstraction: either a triage score was not obtained, or, in some cases when the score was obtained, it was absent from the chart. In addition, even when a triage score was present, there still is no universally accepted standard of triage in the US. In many cases, EDs may use fewer than five triage levels; in others the identical levels in different systems may have different definitions or applications. We attempted to overcome these limitations by deriving ESI-N, a severity index based primarily on the patient’s RFV codes, modified by values in a small number of other data fields, including age, vital sign extremes, and resuscitative procedures. Finally, we separated the less acute visits by number of resources used, emulating the previously derived and validated ESI.^2^

The American College of Emergency Physicians (ACEP) and the Emergency Nurses Association (ENA) formed a task force in 2002 dedicated to the goal of creating and promoting a standard measure of presenting patient acuity.^21^ However, to this day, a number of EDs continue to use locally designed triage guidelines of varying complexity and evidence quality or do not perform formal triage.^22^ In fact, this continued lack of a standard resulted in the ACEP and ENA revising its position statement and advocating for “implementing a standardized emergency department (ED) triage scale and acuity categorization process” and endorsing ESI as that process.^23^

While ESI remains a prominent triage tool, it does not always adequately reflect resource need in linear fashion. That is, ESI Level 1s do not always necessitate the greatest resources in the ED, with ESI 2s requiring less, and so on. In fact, one paper found that ESI Level 2 and 3 patients are actually very similar in their resource needs, but hospitalization varied dramatically.^24^ In contrast, we found positive associations between ESI-N severity and hospital admission, ICU admission, and ambulance transport.^2^ Unlike one prominent early study,^22^ we found that patients who left without being seen were much more likely to have a low severity by ESI-N, a change that could reflect increased use of the ED as a “safety net” rather than for emergencies. This is congruent to findings of some more recent studies.^25^–^26^

Waiting time is often cited as an indicator of ED quality that depends both on hospital capacity as well as the average ED volume and acuity of visits. Recent studies suggest that ED crowding has increased waiting times even for serious problems.^27^ But excluding triage category 1, the ESI-N index discriminates poorly, possibly in part because the nurse assigning a triage category also controls the patient’s waiting time.

## LIMITATIONS

While the ESI-N derived here appears to be a consistent, valid measure of acuity and complexity, there are limits on its use. It is complex: Creating the index requires an algorithm executed by a computer program. It is derived from the 2010–2015 NHAMCS-ED survey files, which were formatted in a specific way; however, variable names and values, such as the RVF classification scheme, have changed and will continue to change over time. Use of the algorithm in survey years other than 2010–2015 may require its modification. Like the ESI itself, devising the ESI-N algorithm required subjective judgment. The index reflects two separate dimensions of ED visits: acuity and complexity. Distinguishing between these dimensions is impossible when evaluating aggregate data. In this paper we describe and validate the ESI-N. It has not been validated and tested for reliability.

## CONCLUSION

Two technical tasks commonly required in health services research include accounting for confounding of a causal relationship, and adjusting for case-mix to minimize selection bias when comparing groups.^28^ Our derivation of a five-level severity index for data abstracted from ED charts addresses these research needs. The ESI-N can be used to stratify results, or as an ordinal exposure or outcome variable in regression or propensity score models, increasing statistical power by reducing the need to include multiple covariates in a model. Caution should be exercised in its application. While it includes observations lost with the use of IMMED, it is less predictive of ED waiting time. It might be used together with IMMED to reduce residual confounding in some analyses. It is important to understand the origin of the index in complaint codes modified by age and a few other variables, and that it segregates less acute visits based on a list of specified services performed. Since this list is short, and since some of the data abstracted are relatively nonspecific (eg, five-level instead of 10-level pain scores), the ESI-N will misclassify some patients compared to a prospectively determined ESI. Future directions include validating ESI-N on another independent source of ED visits and testing its use in a prospective cohort of ED visits.

## Figures and Tables

**Table 1:** Illustrates in detail the procedure we used to derive Emergency Severity Index levels, specifying variable names and values taken from naming conventions in NHAMCS-ED* input programs for public use files.^18^

Patient conditions	Variable name	Occurrences
Level 1		
Dead on arrival (RFV code)	RFV1-RFV3	12
Respiratory arrest	RFV1-RFV3	17
Cardiac arrest	RFV1-RFV3	140
Cardiopulmonary arrest	RFV1-RFV3	21
Unconscious on arrival	RFV1-RFV3	860
Dead on arrival (checkbox)	DOA	50
Pulse ≤50 and age >25	PULSE; AGE	793
Endotracheal intubation	ENDOINT	373
Cardiopulmonary resuscitation	CPR	225
Systolic blood pressure ≤80 and Age >25	BPSYS; AGE	324
Any of level 1 criteria		2,365
% of total weighted (95% CI)	1.4 (1.3, 1.5)	
Level 2 (if not in level 1)		
Fainting (Syncope)	RFV1-RFV3	1,689
Hostile behavior	RFV1-RFV3	536
Neurological weakness or speech difficulty	RFV1-RFV3	488
Shortness of breath/breathing problem	RFV1-RFV3	10,339
Gastrointestinal bleeding	RFV1-RFV3	109
Retention of urine	RFV1-RFV3	472
Sepsis, septicemia	RFV1-RFV3	32
Ischemic heart disease	RFV1-RFV3	100
Violence/self-harm	RFV1-RFV3	1,277
Rape	RFV1-RFV3	99
Altered level of consciousness	RFV1-RFV3	162
Abdominal pain (elderly)	RFV1-RFV3; AGE	144
Abdominal pain, vomiting and diarrhea	RFV1-RFV3	43
Abdominal pain (youth)	RFV1-RFV3; AGE	391
Head Trauma (infants)	RFV1-RFV3; AGE	113
Level 3 exceeding vital sign thresholds	AGEDAYS; PULSE; TEMPF	22
	AGEDAYS; AGE; PULSE	564
	AGE; PULSE	879
	AGE; PULSE	26,814
Any of level 2 criteria		40,178
% of total weighted (95% CI)	24.5 (24.1, 25.0)	
Level 3 (if not in level 1–2)		
More than 1 resource used	(see Resources below)	53,704
Severe pain	PAINSCALE	29,291
Pediatric fever	AGE; TEMPF	785
Motor vehicle accident		2,044
Any of level 3 criteria		70,230
% of total weighted (95% CI)	42.5 (41.7, 43.3)	
Level 4 (if not in level 1–3)		
One resource used	(see Resources below)	24,974
% of total weighted (95% CI)	15.1 (14.6, 15.7)	
Level 5 (if not in level 1–4)		
No resource used	(see Resources below)	27,408
% of total weighted (95% CI)	16.4 (15.7, 17.1)	

* *NHAMCS-ED*, National Hospital Ambulatory Medical Care Survey-emergency department visits.

*RFV*, reason for visit; *CI*, confidence interval.

**Table 2:** Mean ESI-N for patient characteristics.

Patient characteristics	Weighted patient # (in 1000s)	Mean ESI-N level (95% CI)
All Visits	805,726	3.21 (3.19, 3.23)
Gender		
Female	445,253	3.15 (3.13, 3.17)
Male	360,473	3.27 (3.25, 3.29)
Age		
Under 15 years	152,469	3.76 (3.72, 3.79)
15–24 years	124,430	3.20 (3.18, 3.23)
25–44 years	227,839	3.12 (3.10, 3.15)
45–64 years	176,474	3.05 (3.03, 3.07)
65–74 years	54,185	2.94 (2.91, 2.97)
75 years and over	70,328	2.88 (2.86, 2.91)
Race		
Non-Hispanic White	476,805	3.15 (3.13, 3.18)
Non-Hispanic Black	180,130	3.26 (3.23, 3.29)
Hispanic	124,909	3.33 (3.30, 3.36)
Non-Hispanic other	23,882	3.23 (3.18, 3.28)
Expected source of payment		
All sources of payment are blank	10,470	3.37 (3.26, 3.48)
Unknown	48,878	3.25 (3.20, 3.30)
Private insurance	230,145	3.22 (3.19, 3.24)
Medicare	146,598	2.93 (2.90, 2.95)
Medicaid or CHIP	227,873	3.35 (3.32, 3.37)
Worker’s compensation	6,857	3.57 (3.49, 3.65)
Self-pay	105,473	3.22 (3.19, 3.25)
No charge/charity	7,113	3.15 (3.08, 3.23)
Other	22,320	3.19 (3.14, 3.24)
Region		
Northeast	140,858	3.25 (3.21, 3.29)
Midwest	187,086	3.19 (3.15, 3.24)
South	310,329	3.19 (3.16, 3.22)
West	167,453	3.21 (3.17, 3.25)
Visit Year		
2010	129,843	3.19 (3.16, 3.23)
2011	136,296	3.17 (3.14, 3.21)
2012	130,870	3.21 (3.17, 3.24)
2013	130,353	3.22 (3.18, 3.25)
2014	141,420	3.25 (3.20, 3.29)
2015	136,943	3.21 (3.15, 3.26)

*ESI-N*, Emergency Severity Index levels based primarily on the patient’s “reason for visit” code as presented in the National Hospital Ambulatory Care Survey; *CI*, confidence interval; *CHIP*, Children’s Health Insurance Program.

**Table 3:** Number of emergency department visits by Emergency Severity Index-N levels.

Criterion	Weighted patient # (in 1000s)	ESI-N level				

		Immediate (level 1) (%)	Emergent (level 2) (%)	Urgent (level 3) (%)	Semi-urgent (level 4) (%)	Non-urgent (level 5) (%)
All visits	805,726	1.4 (0.1)	24.5 (0.2)	42.5 (0.4)	15.1 (0.3)	16.4 (0.4)
Admitted to hospital	83,607	4.7 (0.2)	40.6 (0.5)	49.2 (0.7)	3.4 (0.2)	2.2 (0.6)
Admitted to critical care unit	10,875	15.0 (1.1)	46.3 (1.4)	35.9 (1.3)	1.8 (0.3)	*
Arrived by ambulance	122,246	5.0 (0.2)	33.1 (0.5)	48.1 (0.6)	8.2 (0.3)	5.5 (0.4)
Left without being seen	8,012	*	28.4 (1.4)	41.4 (1.7)	9.5 (1.2)	19.4 (1.3)

* Figure does not meet standard or reliability of precision.

*ESI-N*, Emergency Severity Index levels based primarily on the patient’s “reason for visit” code as presented in the National Hospital Ambulatory Care Survey.

**Table 4:** Rates of hospital admission by ESI-N levels.

Criterion	ESI-N level (Weighted patient # [in 1000s], %)									

	Immediate (level 1)		Emergent (level 2)		Urgent (level 3)		Semi-urgent (level 4)		Non-urgent (level 5)	
Admitted to hospital	3,425	37.9% (34.5, 41.2)	28,981	18.0% (16.8, 19.2)	34,117	12.3% (11.4, 13.2)	2,274	2.5% (2.1, 2.9)	1,504	1.5% (0.6, 2.4)
Length of stay	201.4 (191.3, 211.5)		180.2 (174.2, 186.3)		182.7 (177.2, 188.3)		119.5 (115.4, 123.5)		82.9 (80.0, 85.8)	
Waiting time	13.5 (11.8, 15.2)		22.5 (21.2, 23.8)		24.4 (23.0, 25.8)		24.5 (22.6, 26.3)		24.9 (23.3, 26.5)	

*ESI-N*, Emergency Severity Index levels based primarily on the patient’s “reason for visit” code as presented in the National Hospital Ambulatory Care Survey.
